# Large-scale causal analysis of gut microbiota and six common complications of diabetes: a mendelian randomization study

**DOI:** 10.1186/s13098-024-01298-9

**Published:** 2024-03-13

**Authors:** Jiachen Wang, Menghao Teng, Ruoyang Feng, Xiaochen Su, Ke Xu, Junxiang Wang, Guoqiang Wang, Yulong Zhang, Peng Xu

**Affiliations:** 1 Department of Joint Surgery, HongHui Hospital, Xi’an Jiaotong University, 710054 Xi’an, Shaanxi China; 2 Department of Orthopedics, The First Affiliated Hospital of Xi’an Jiaotong University, 710054 Xi’an, Shaanxi China

**Keywords:** Complications of diabetes, Gut microbiota, Mendelian randomization study, Genome-wise association studies, Single-nucleotide polymorphism

## Abstract

**Background:**

This study aimed to reveal the association between the gut microbiota (GM) and six diabetic complications: diabetic hypoglycemia; ketoacidosis; nephropathy; neuropathy; retinopathy; and Charcot’s foot.

**Methods:**

GM data were obtained from the MiBioGen consortium and Dutch Microbiome Project while data on the six diabetic complications were obtained from the FinnGen consortium. Two-sample Mendelian randomization (TSMR) was performed to explore the association between GM and the common diabetic complications. Inverse MR analysis was conducted to examine the effect of diabetic complications on the identified GM. Sensitivity tests were conducted to validate the stability of the results. Finally, multivariate MR (MVMR) was performed to determine whether GM had a direct influence on the diabetic complications.

**Results:**

After multiple corrections, the inverse variance weighted (IVW) results predicted 61 suggestive markers between GM and six diabetic complications. In particular, the IVW results revealed that the Bacteroidia class and Bacteroidales order were positively associated with diabetic hypoglycemia while the Verrucomicrobiae class and Verrucomicrobiales order were positively associated with diabetic nephropathy. Based on the replication analysis, these results were identified to be stable. MVMR showed that the results remained stable after accounting for traditional risk factors.

**Conclusion:**

Extensive causal associations were found between GM and diabetic complications, which may provide new insights into the mechanisms of microbiome-mediated complications of diabetes.

**Supplementary Information:**

The online version contains supplementary material available at 10.1186/s13098-024-01298-9.

## Introduction

Diabetes is a chronic metabolic disease characterized by elevated blood glucose levels [1]. According to the World Health Organization, the incidence of diabetes has been increasing annually. As a result, approximately 629 million adults are expected to develop diabetes by 2045 [2]. As balancing the blood glucose level is difficult, patients with diabetes often develop serious complications. Approximately 30% of patients experience diabetic kidney disease, 35% of patients may develop varying degrees of diabetic retinopathy [3], and 34% of patients will experience different levels of peripheral neuropathy [4]. Although the precise mechanisms underlying these complications remain unclear, chronic inflammation, oxidative stress, hypertension, and dyslipidemia are widely recognized as potential risk factors [5, 6]. These complications affect the quality of life of patients with diabetes, leading to severe health issues, such as disability, organ failure, and even death. In addition, long-term treatment and medical management of these complications result in marked economic and healthcare burdens on individuals and society [7].

The gut microbiota (GM) are the microorganisms that reside in the human intestine and interact with the human body [8]. The GM exhibit various physiological effects on the body and are involved in the synthesis of enzymes and vitamins, and the digestion and absorption of three essential nutrients [9]. In addition, the GM play a role in the maintenance of the intestinal barrier integrity and resistance of pathogenic bacteria invasion by forming a protective barrier on the surface of the intestinal mucosa [10]. Notably, the GM regulate the development and function of immune cells and maintain the balance and stability of the immune system [11]. Based on sufficient evidence, the GM plays a role in the development of diabetes from multiple aspects [12, 13]. In recent years, several studies found associations between the GM and diabetic complications. In particular, Zhang et al. found a decrease in the abundance of *Clostridium*, *Eubacterium*, *Roseburia intestinalis*, *Lachnospira*, and *Intestinibacter* and an increase in the abundance of *Bacteroidetes faecalis* in patients with diabetic kidney disease [14]. Huang et al. revealed an increase in the abundance of *Bifidobacterium* and *Lactobacillus*, along with a decrease in the abundance of *Escherichia-Shigella*, *Faecalibacterium*, *Pasteurellaceae*, and *Clostridium* in patients with diabetic retinopathy [15]. However, these studies were retrospective in nature and may have been biased by multiple confounding factors and potential reverse causality. For example, individuals with diabetes always require diet changes, treatment with medication, and physical exercise. The interaction between GM and anti-diabetic drugs, the interaction between GM and daily diet, and the impact of exercise on GM have been previously investigated [16–18]. These confounding factors (such as diet, medication, and physical exercise) also influence the development of diabetic complications [19]. As previous studies on the causal association between GM and diabetic complications are affected by these confounders, genetic evidence is urgently needed.

Mendelian randomization (MR) is an epidemiological research method used to investigate the mechanisms of disease occurrence. MR uses single-nucleotide polymorphisms (SNPs) as instrumental variables (IVs) to infer potential causal effects between risk factors and outcomes [20]. MR can avoid the interference of confounding factors because SNPs are randomly assigned at conception [21]. As the genotype of an individual is irreversible throughout their lifetime, MR can exclude the interference of reverse causation. Currently, the MR method is widely used to identify risk factors of diabetic complications [22, 23]. Owing to the extremely high reliability of previous MR analyses at evaluating causality, MR analysis was conducted to assess the causal associations between the GM and the diabetic complications at the genetic level, which is expected to make a considerable contribution to further research on diabetic complications.

## Materials and methods

### Study design

**Fig. 1:**
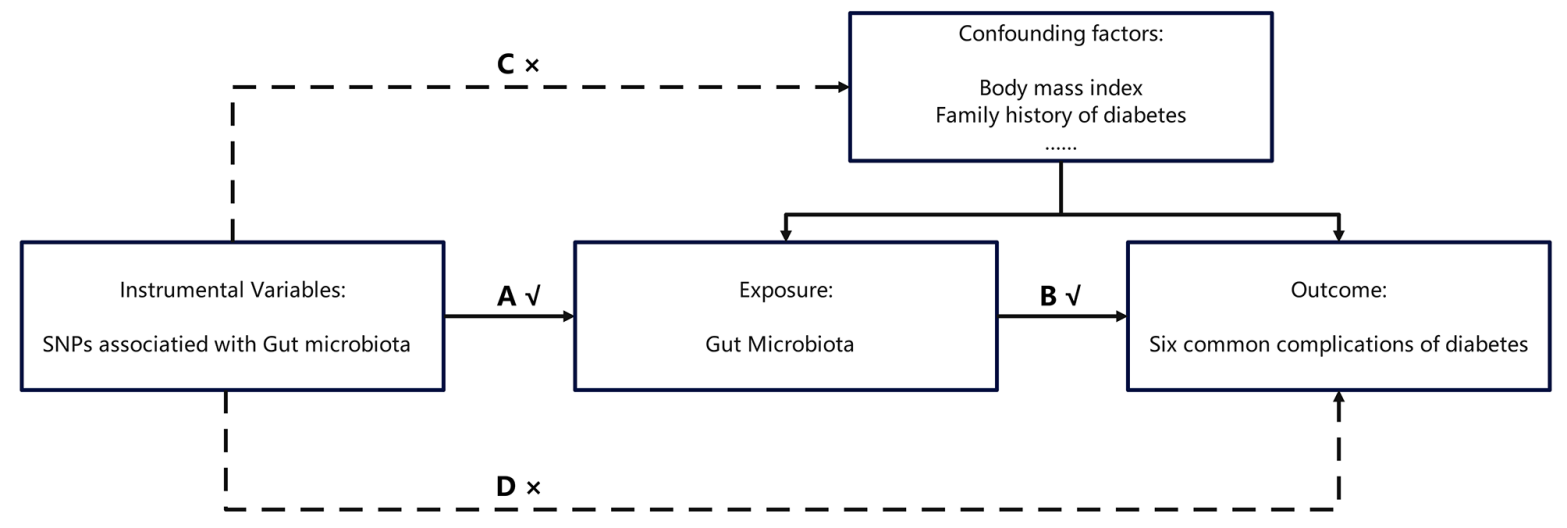
Assumptions that must be met by MR analysis: Single-nucleotide polymorphisms associated with gut microbiota (GM) were initially regarded as instrumental variables (IVs). All the IVs can only associate with GM (the solid line and ‘A’ and ‘B’ are permitted), and the selected IVs should not associate with confounding factors or outcomes directly (the dashed line and arrows ‘C’ and ‘D’ are not allowed)

**Fig. 2:**
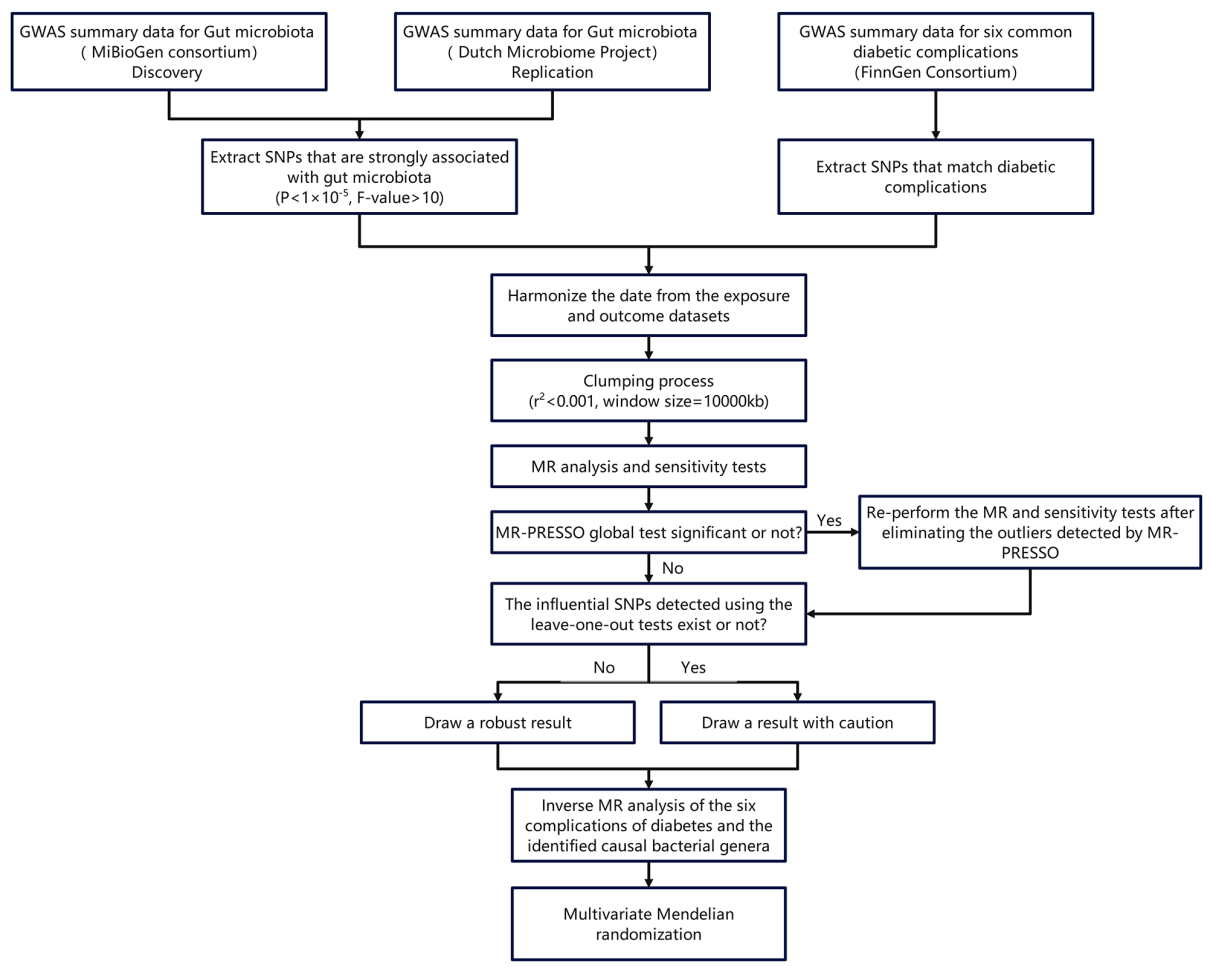
Flow chart of MR analysis: Flow chart of the analysis. Two-sample MR was performed to explore the genetic associations between the gut microbiota and six diabetic complications. Inverse MR analysis was conducted to explore the associations between the diabetic complications and the identified GM. Finally, multivariate MR was performed to determine whether the effect of identified GM taxa on the six diabetic complications were direct or indirect

Two-sample MR (TSMR) was performed to explore the causal genetic associations between GM taxa and six diabetic complications. The data used in our study were obtained from the available genome-wide association studies (GWAS) summary-level data. Rigorously selected SNPs were regarded as IVs for further MR analyses. The MR analysis must meet three requirements: the selected IVs must be associated with exposure; the associations between the selected SNPs and outcomes must be free of confounding factors; and the selected SNPs must not be related to any outcomes other than exposure. The three requirements and a flowchart of the MR analysis are shown in Figs. 1 and 2, respectively. For the primary MR results used to assess the impact of GM on diabetic complications, an inverse MR analysis was performed to determine the effect of diabetic complications on the identified GM. Multivariate MR (MVMR) was then conducted to identify the independent causal effects of GM on diabetic complications after excluding traditional risk factors. The process used for the analysis is illustrated in Fig. 2.

R software (version 4.2.0) and the R packages, “Two-sample MR,” “MVMR,” and “MR-PRESSO,” were used to perform the statistical analyses. Statistical significance was set at *P* < 0.05. As publicly available abstract data were employed in this study, ethical approval was not required.

### Data resource

Summary data of the six common diabetic complications were obtained from the FinnGen consortium which is based on a combination of genomic information and digital healthcare data from national registries. The 9th version FinnGen Study (https://r9.finngen.fi/), released in May 2023, was employed in this study. In the 9th version, 377,277 samples were included, and up to 20,175,454 variants were analyzed. The six common diabetic complications, including diabetic hypoglycemia (7,332 cases and 271,817 controls), diabetic ketoacidosis (7,841 cases and 271,817 controls), diabetic nephropathy (4,111 cases and 308,539 controls), diabetic neuropathy (2,843 cases and 271,817 controls), diabetic retinopathy (6,818 cases and 344,569 controls), and Charcot’s foot (473 cases and 271,817 controls), were regarded as outcomes in our study (Supplementary Table 1). All patients in the FinnGen project underwent strict quality control procedures and were genotyped using Illumina (Illumina) and Affymetrix arrays (Thermo Fisher Scientific). Age, sex, the first 10 principal components, and genotyping batch were corrected during the analysis; detailed information can be found elsewhere [24].

Summary data on GM composition were obtained from the MiBioGen consortium and Dutch Microbiome Project (DMP). The data from MiBioGen consortium was used as discovery datasets, and it includes a summary of the analysis of 18,340 participants from 24 cohorts in Germany, Canada, the USA, Israel, Finland, South Korea, Belgium, Denmark, the Netherlands, Sweden, and the UK. 16 S rRNA gene sequencing profiles and genotyping results were derived from this analysis. Up to 131 genera, 35 families, 20 orders, 16 classes, and 9 phyla with a mean abundance higher than 1% were included in the study. The summary data on GM composition from the DMP were used as candidate replication datasets to assess the reliability of the identified significant causal associations. In DMP, a total of 207 taxa and 205 bacteria metabolism pathways were analyzed in 7,738 participants of European ancestry. More detailed information on the GWAS summary data of the GM used in the present study can be found in the previously published studies [25, 26].

### Selection of IVs

The SNPs that underwent strict selection were considered IVs. First, genome-wide significance (*p* < 5 × 10^− 8^) was used to select SNPs associated with GM taxa. However, the number of selected SNPs was relatively small below this threshold. The significance threshold was set at *P* < 1 × 10^− 5^. Thereafter, the PLINK aggregation method was used to calculate linkage disequilibrium among the SNPs; SNPs with r^2^ > 0.001 and a base physical distance of less than 10 000 kb were removed. Finally, to minimize the influence of weak instrumental bias, SNPs with F-values of < 10 were excluded. The following equation was used to calculate the Fvalue:

$$ \mathbf{F}={\mathbf{R}}^{2}\frac{\left(\mathbf{N}-2\right)}{\left(1-{\mathbf{R}}^{2}\right)}$$

where $\text{N}$ is the sample size and R^2^ is the genetic variation. The following equation was used to calculate R^2^:

$$ {\mathbf{R}}^{2}=2\times \mathbf{E}\mathbf{A}\mathbf{F}\times \left(1-\mathbf{E}\mathbf{A}\mathbf{F}\right)\times {\beta }^{2}$$

where EAF is the effect allele frequency and β is the allele effect value.

PhenoscannerV2 was used to eliminate the SNPs associated with outcomes and confounding factors (*P* < 1  × 10^− 5^). PhenoscannerV2 is an effective tool for exploring the association between human genotypes and phenotype. Traditional risk factors for diabetic complications, including body mass index (BMI) and family history of diabetes, and damage to pancreatic beta cells caused by viral infections (such as rubella, mumps), were regarded as confounding factors in our study [27–29]. Finally, we harmonized the effect allele of exposure and outcome data, and removed palindromic SNPs with intermediate allele frequencies. For inverse MR, the selection of instrumental variables was consistent with the abovementioned steps, and the genome-wide significance (*p* < 5 × 10^− 8^; *p* < 5 × 10^− 6^) was used to select SNPs associated with six complications of diabetes.

### Statistical analysis

TSMR was performed to explore the causal associations between GM and the six chronic complications of diabetes. The following five methods were employed to obtain the MR estimates: MR Egger, Weighted median, inverse variance weighted (IVW), constrained maximum likelihood-based MR (Cml-MA), and weighted mode methods. IVW combines the ratio estimates of each SNP using meta-analysis and provides the most precise estimate in the absence of horizontal pleiotropy and heterogeneity. Therefore, in this study, IVW was regarded as the primary method [30]. The MR Egger method is similar to the IVW method, and combines the ratio estimates of each SNP using meta-regression and obtains the estimates after adjusting for directional pleiotropy. Therefore, although all SNPs used in MR Egger were invalid IVs, a relatively precise estimate was obtained [31]. The weighted median model can obtain consistent estimates of causal effects when up to 50% of the SNPs used in the study are invalid IVs [32]. Furthermore, Cml-MA, which is based on constrained maximum likelihood and model averaging methods, is robust against invalid IVs with irrelevant or relevant pleiotropic effects. Therefore, Cml-MA can better control the Type-I error rate in the estimation [33]. Bonferroni correction (0.05/n, where n is the number of bacterial taxa included in each level) was applied for multiple-testing correction. The significance threshold for five taxa levels was set as follows: genera *P* = 3.82 × 10^–4^ (0.05/131), families *P* = 1.43 × 10^–3^ (0.05/35), orders *P* = 2.50 × 10^–3^ (0.05/20), classes *P* = 3.13 × 10^–3^ (0.05/16), and phyla *P* = 5.56 × 10^–3^ (0.05/9). For the primary TSMR results of the effect of GM on the complications of diabetes, inverse MR analysis was performed to explore the associations between the six complications of diabetes and the identified causal bacterial genera.

Various methods were employed to test the sensitivity of these results. Cochran’s Q test was performed to assess the heterogeneity of the results. Cochran’s Q test uses the IVW method and the MR Egger test to obtain the Q-value. A Q-value that is markedly higher than the degrees of freedom suggests the existence of heterogeneity. Egger intercept and MR-PRESSO global tests were conducted to determine the global horizontal pleiotropy of the association. The MR-PRESSO outlier test was conducted to detect the outliers existing in the associations; thereafter, correction for horizontal pleiotropy was performed. The MR-PRESSO distortion test was performed to determine whether the causal association was significantly distorted before and after outlier removal. Finally, a leave-one-out test was conducted to determine whether a single influential SNP affected the estimates.

The abundance of different GM is affected by lifestyle and physical conditions. A simple TSMR analysis cannot reflect the direct effect of GM on the complications of diabetes because living habits and physical conditions can also affect the development of diabetic complications [34]. Therefore, multivariate MR (MVMR) was performed to determine whether the effect of identified GM taxa on the six complications of diabetes were direct or indirect [35].

## Results

### Selection of IVs

In discovery datasets, as the names of 15 bacterial taxa were not available, 196 bacterial taxa (9 phyla, 16 classes, 20 orders, 32 families, and 119 genera) were included in the MR study. The number of IVs included in these bacterial taxa ranged from 2 to 20, as shown in Supplementary Table 2. All F-values of the SNPs used in our MR analysis were greater than 10, which indicates that the influence of weak instrumental bias is less likely (Supplementary Table 2). In the inverse analysis, the IVs of the six complications of diabetes were 2–17.

### Two-sample MR results

**Fig. 3:**
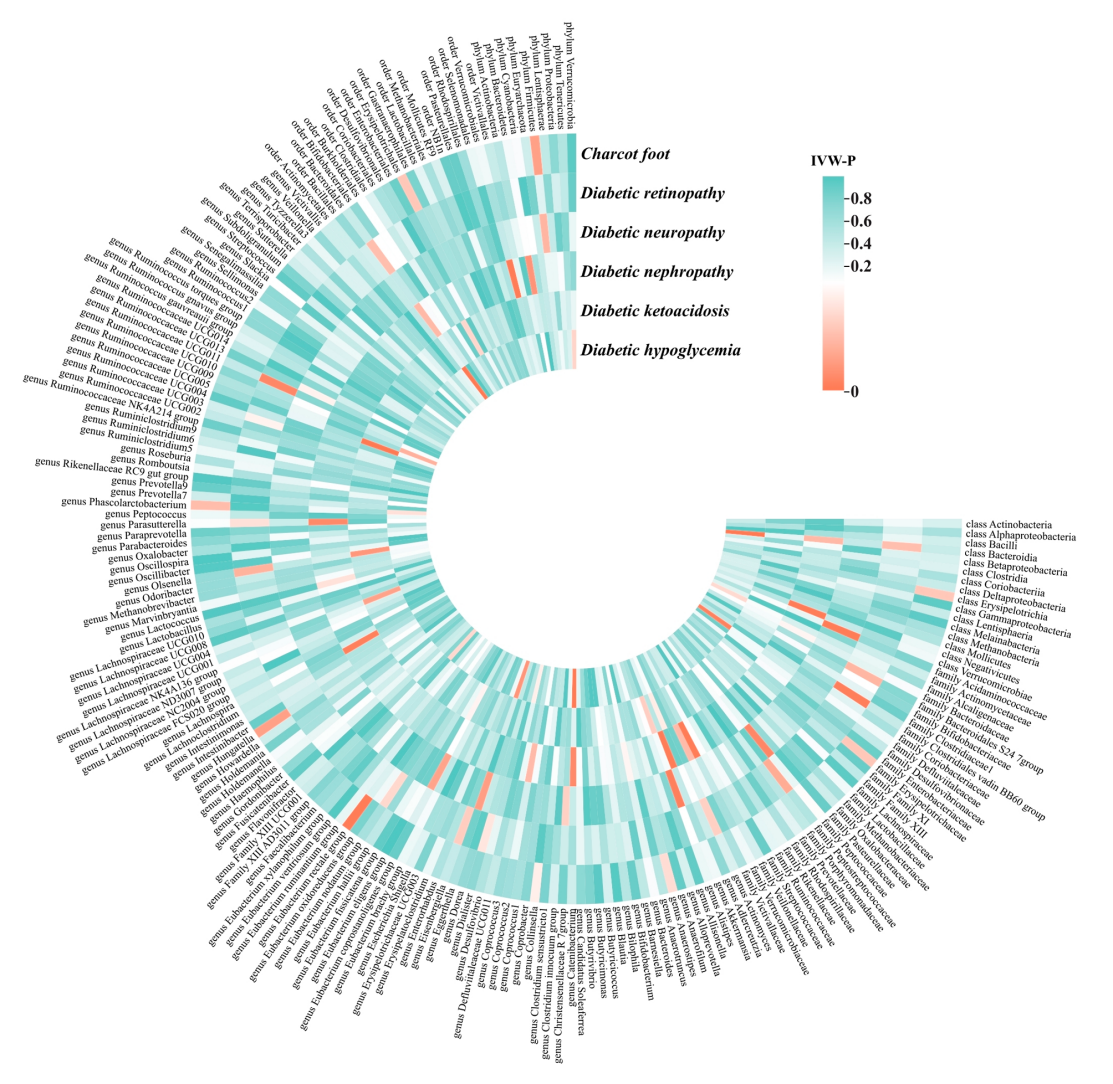
IVW results of the causal association between the gut microbiota and the six common diabetic complications

**Fig. 4:**
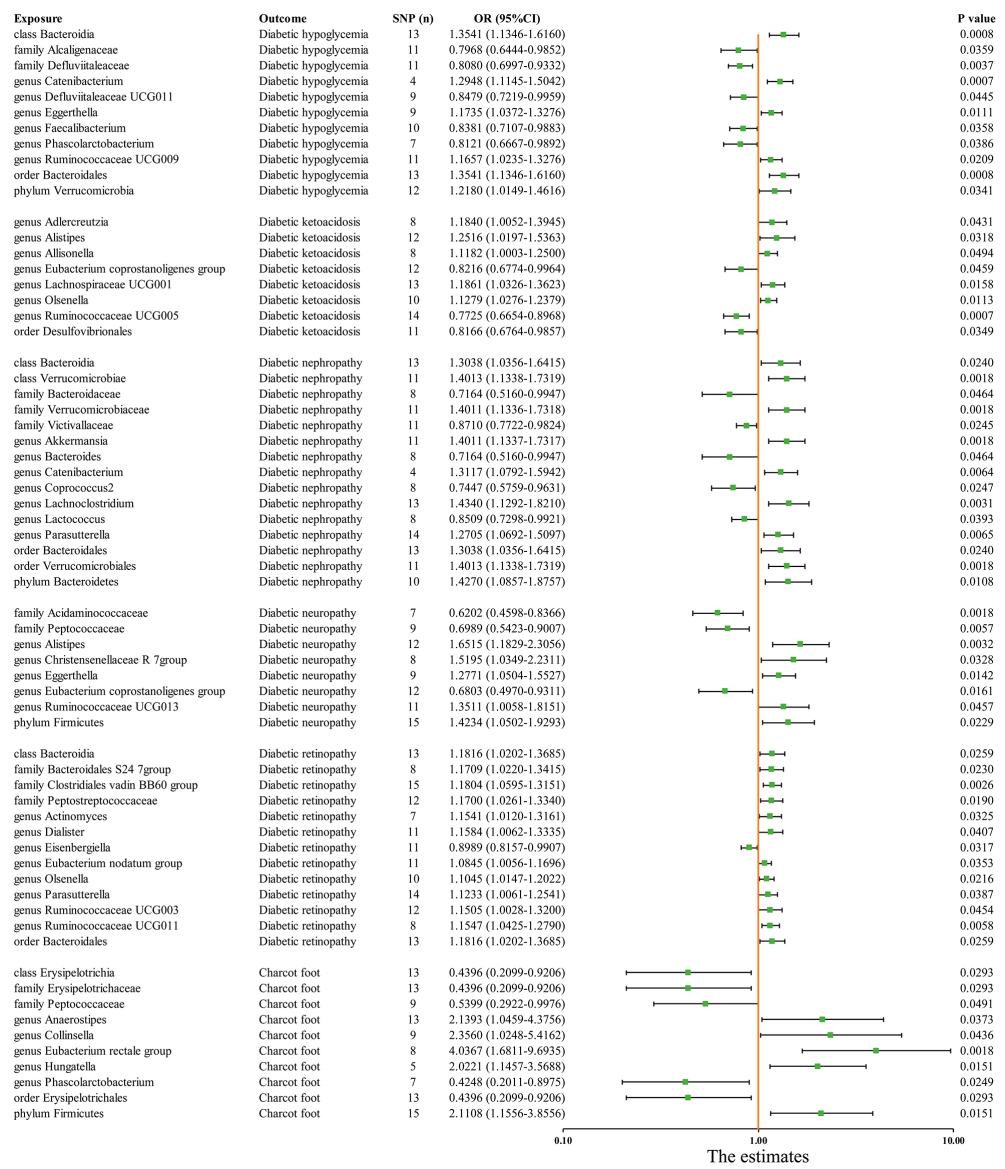
Significant results between the gut microbiota and the six common complications of diabetes

The IVW results from MiBioGen consortium genetically predicted extensive causal associations between GM and six diabetic complications (Fig. 3 and Supplementary Table 3). All significant results are shown in Fig. 4 and provide suggestive evidence of the effect of GM on the complications of diabetes. After multiple testing, the IVW results revealed that the Bacteroidia class and Bacteroidales order were independent risk factors for diabetic hypoglycemia (class and order: OR = 1.3541, 95% confidence interval (CI): 1.1346–1.6160, P-IVW = 0.0008), whereas the Verrucomicrobiae class and Verrucomicrobiales order were positively associated with diabetic nephropathy (class: OR = 1.4013, 95% CI: 1.1338–1.7319, P-IVW = 0.0018; order: OR = 1.4011, 95% CI: 1.1336–1.7318, P-IVW = 0.0018). In the inverse MR analysis, the IVW method revealed that the six common complications of diabetes had a null effect on the identified taxa of GM (Supplementary Table 4).

In the replication analysis stages. For four significant associations. The IVW results from the DMP revealed that these associations are in the same direction with those of the discovery analysis. Furthermore, the IVW results showed *Paraprevotella*, *Prevotella*, and *Alistipes* genera in the Bacteroidales order were positively associated with diabetic hypoglycemia, while MR-Egger results showed *Akkermansia* genera in the Verrucomicrobiales order was positively associated with diabetic nephropathy (Supplementary Table 5). For the remaining 61 suggestive associations, the replications analysis revealed directional consistency for most associations (Supplementary Table 5).

The Cochran’s Q test revealed that heterogeneity did not exist in these associations. The MR Egger intercept and MR-PRESSO global tests revealed no evidence of the existence of horizontal pleiotropy, and MR-PRESSO did not detect any outliers. The results of the sensitivity tests are presented in Supplementary Table 6. The leave-one-out tests revealed that the significant results are stable and robust (Supplementary Fig. 1). The funnel plots also revealed symmetrical distribution of the SNPs (Supplementary Fig. 2).

### Multivariate MR results

**Fig. 5:**
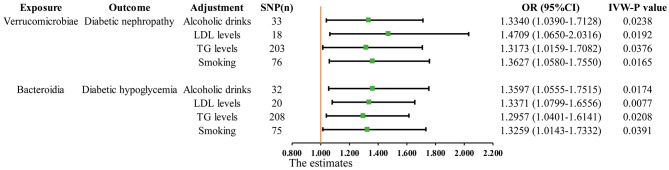
Multivariate MR results: After adjusting for confounding factors, including consumption of alcoholic drinks, LDL levels, TG levels, and smoking, the significant results remained stable

MVMR was performed to determine whether the effect of GMs on the six common complications of diabetes was direct or was due to common living habits or physical conditions, such as tobacco intake, alcohol consumption, TG, and LDL. After adjusting for these confounding factors, the Bacteroidia class and Bacteroidales order remained positively associated with diabetic hypoglycemia, and the Verrucomicrobiae class and Verrucomicrobiales order were positively associated with diabetic nephropathy **(**Fig. 5**)**.

## Discussion

This study sought to systematically evaluate the causal associations between GM and six common diabetic complications (diabetic retinopathy, neuropathy, nephropathy, ketoacidosis, hypoglycemia and Charcot’s foot). SNPs were rigorously selected as IVs from the largest GWAS of the GM, and outcome data were obtained from the FinnGen Consortium. Finally, a total of 65 causal associations were found, four of which were strongly correlated and 61 were nominally correlated, highlighting the importance of GM in diabetic complications.

Based on accumulating evidence, a significant association exists between the development of diabetes and its complications and changes in the composition of the GM. For example, Larsent et al. found that the ratio of Bacteroidetes to Firmicutes phylum is positively associated with serum glucose levels; the abundances of butyrate-producing bacteria, such as *Faecalibacterium prausnitzii* and *Eubacterium rectale*, are decreased, while the abundance of *Lactobacillus* species is increased in patients with diabetes [36]. Wang et al. found an increase in the abundance of Firmicutes and Actinobacteria and a decrease in the abundance of Bacteroidetes in patients with diabetic neuropathy [37]. Compared to healthy controls, the abundance of Bacteroidetes, Mucoromycota, and Pasteurellaceae was decreased and the proportions of *Actinobacteria*, *Acidaminococcus*, *Escherichia*, and *Enterobacter* were significantly increased in patients with diabetic retinopathy [15, 38–41]. According to a Chinese study, mice with diabetic nephropathy have a reduced abundance of Firmicutes and an increased abundance of *Allobaculum* and *Anaerosporobacter* [42]. Although the association between GM and various diabetic complications is not well defined, certain metabolites produced by the GM, such as amino acids, trimethylamine N-oxide, bile acids, and indolepropionic acid, are involved in the regulation of host metabolism and gut integrity, ultimately contributing to the occurrence of diabetes and its complications [43, 44]. Overall, most previous studies on GM and diabetic complications have been observational. Thus, the present study is the first to provide genetic evidence of the association between GM and six diabetic complications.

The Bacteroidia class and Bacteroidales order were associated with an increased risk of diabetic hypoglycemia. Diabetic hypoglycemia is a frequent and serious complication among patients with diabetes mellitus. Diabetic hypoglycemia is characterized by glucose levels < 4 mmol/L and often clinically manifests as symptoms of sympathetic excitation, such as palpitations, fatigue, hunger, and cerebral cortex suppression, including drowsiness and unconsciousness in severe cases [45, 46]. Bacteroidales is a potentially colonizing bacterium of the colon that accounts for 25% of the GM [47, 48]. According to some studies, Bacteroidales species are relatively less abundant in the GM of individuals with obesity and the relative abundance of Bacteroidales increases as the weight of individuals with obesity decreases, which may be associated with carbohydrate metabolism [49]. Previous studies have revealed that interfering with the gut ecosystem using Bacteroidales can increase the activity of activated protein kinases and modulate the transmission of inflammatory signals within the intestines, thereby augmenting insulin-stimulated glucose transport in muscles, which results in improved insulin sensitivity and glucose tolerance [50]. Accordingly, we speculate that Bacteroidales can affect intestinal inflammatory signaling, thereby inhibiting the digestion and absorption of intestinal polysaccharides, while promoting glucose transport and metabolism in the skeletal muscle, ultimately leading to diabetic hypoglycemia.

The Verrucomicrobiae class and the Verrucomicrobiales order were found to be potential risk factors for diabetic nephropathy. Diabetic nephropathy is a microvascular complication of diabetes characterized by glomerular sclerosis, tubular hypertrophy, basement membrane thickening, and interstitial fibrosis in the kidneys [51]. Diabetic nephropathy occurs in 40% of patients with diabetes, is primarily induced by chronic hyperglycemia, and is a major cause of chronic kidney disease [52]. The pathogenesis of diabetic nephropathy is extremely complex and involves interactions between various pathways. In patients with diabetes, hyperglycemia, high lipid levels, oxidative stress, and reactive oxygen species can damage kidney cells and activate proinflammatory signaling pathways [51]. In addition, glycated proteins can directly activate the complement system and proinflammatory signaling [53]. In response to the sustainable activation of innate immunity, mesangial cells, endothelial cells, and podocytes produce a series of inflammatory mediators, such as chemokines, cytokines, and adhesion molecules. These inflammatory mediators activate and recruit monocytes and macrophages, leading to a further inflammatory cascade [54]. Ultimately, sustainable chronic inflammation leads to remodeling of the kidney structure and tubulointerstitial fibrosis [50, 54]. Verrucomicrobiae is a beneficial bacterium widely distributed in the gastrointestinal tract of healthy individuals that can regulate inflammatory processes [55]; a decrease in the abundance of Verrucomicrobiae indicates an imbalance in the gut microbial ecosystem [56]. In many studies, a reduced abundance of Verrucomicrobiae was found in individuals with type 2 diabetes [57]. However, another study found that an increase in the abundance of Verrucomicrobiae contributes to the progression of type 2 diabetes in mice, which might be related to a systemic low-grade inflammatory response induced by positive feedback mechanisms [58]. As a result, we speculate that in patients with diabetes, the increased abundance of Verrucomicrobiae causes a systemic inflammatory response, which promotes structural and functional abnormalities of the kidneys, leading to the occurrence of diabetic nephropathy.

Our study provides genetic evidence of the extensive causal associations between GM and diabetic complications. The use of MR methods and various sensitivity tests minimized the influence of confounding factors and reverse causality, which always exist in a traditional retrospective study. In the present study, we revealed the dual role of GM in diabetic complications, which provide a new perspective for the combined treatment of diabetic complications by the GM and traditional drugs. Due to individual differences among patients with diabetes and short-term changes in GM, only a small part of the studies on GM in diabetic complications can be translated into clinical practice [59]. Our study can provide new genetic targets for dietary therapy, probiotic and engineered microbial therapies, and fecal microbial transplantation for the treatment of diabetic complications.

To our knowledge, this is the first MR study to explore the potential genetic associations between the GM and the various diabetic complications. This study had several strengths. First, the causal associations were not influenced by the bias caused by confounders or reverse causality. Moreover, the SNPs were rigorously selected from different large GWAS summary datasets, and the cases underwent strict quality control in these summary data. Finally, various methods were used to evaluate the sensitivity of the associations, validating the reliability and stability of the results.

The current MR studies had several limitations. All data were obtained from individuals of European ancestry, thereby serving as the primary limitation of the study. Further studies are required to apply our results to other populations. Owing to the characteristics of the GWAS summary data, we could not conduct subgroup analysis based on factors, such as age and sex. Of note, horizontal pleiotropy is inevitable in MR analysis. Although the Egger intercept MR-PRESSO method was employed to detect the existence of horizontal pleiotropy and MVMR was used to avoid the influence of common living habits, caution should be exercised when applying the results obtained using MR. Finally, our MR study provided extensive genetic evidence of the association between GM and six common diabetic complications. A strong causal correlation was found. Additional suggestive evidence should be further investigated.

## Conclusion

MR was performed to investigate potential genetic associations between GM and the six common complications of diabetes. Several significant associations that elucidate the complex interplay between GM and diabetic complications were found. A strong genetic association was identified between specific bacterial taxa and diabetic complications. The Bacteroidia class and Bacteroidales order were positively associated with hypoglycemia, indicating their potential roles in these complications. In addition, the Verrucomicrobiae class and Verrucomicrobiales order were positively associated with diabetic nephropathy. These findings provide valuable insights into the genetic underpinnings of diabetic complications and highlight the role of the GM in diabetes-related health issues. Overall, this MR analysis enhances our understanding of the genetic links between the GM and diabetic complications, laying the foundation for potential therapeutic interventions and personalized treatment strategies for individuals with diabetes.

### Electronic supplementary material

Below is the link to the electronic supplementary material.

**Supplementary Figure 1**: Leave-one-out test of the significant results: Leave-one-out tests for the associations between the gut microbiota and the six common complications of diabetes. A Bacteroidia class and diabetic hypoglycemia. B Bacteroidales order and diabetic hypoglycemia. C Verrucomicrobiae class and diabetic nephropathy. D Verrucomicrobiales order and diabetic nephropathy

**Supplementary Figure 2**: Funnel plots of the significant results: Funnel plots of the associations between the gut microbiota and six common complications of diabetes. A Bacteroidia class and diabetic hypoglycemia. B Bacteroidales order and diabetic hypoglycemia. C Verrucomicrobiae class and diabetic nephropathy. D Verrucomicrobiales order and diabetic nephropathy

Supplementary Material 3

Supplementary Material 4

Supplementary Material 5

Supplementary Material 6

Supplementary Material 7

Supplementary Material 8

## Data Availability

Data is provided within the manuscript or supplementary information files.
