# Supplementary figures and images for: Large-scale causal analysis of gut microbiota and six common complications of diabetes: a mendelian randomization study
Source: Diabetol Metab Syndr. 2024 Mar 13;16:66. doi: 10.1186/s13098-024-01298-9 (PMC10935953; doi:10.1186/s13098-024-01298-9)

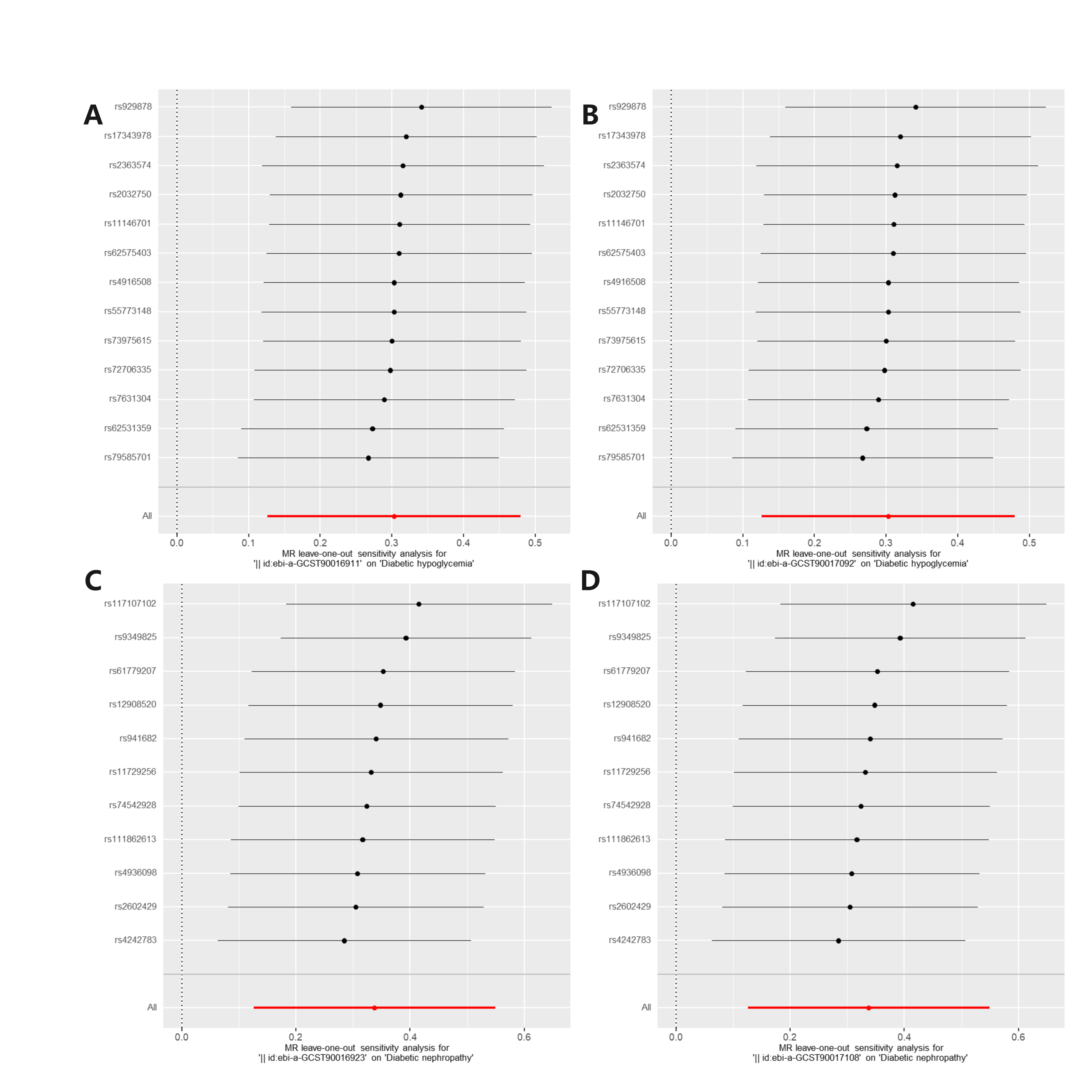

Supplement: Supplementary file 1 — Supplementary Figure 1: Leave-one-out test of the significant results: Leave-one-out tests for the associations between the gut microbiota and the six common complications of diabetes. A Bacteroidia class and diabetic hypoglycemia. B Bacteroidales order and diabetic hypoglycemia. C Verrucomicrobiae class and diabetic nephropathy. D Verrucomicrobiales order and diabetic nephropathy [file 13098_2024_1298_MOESM1_ESM.tif]

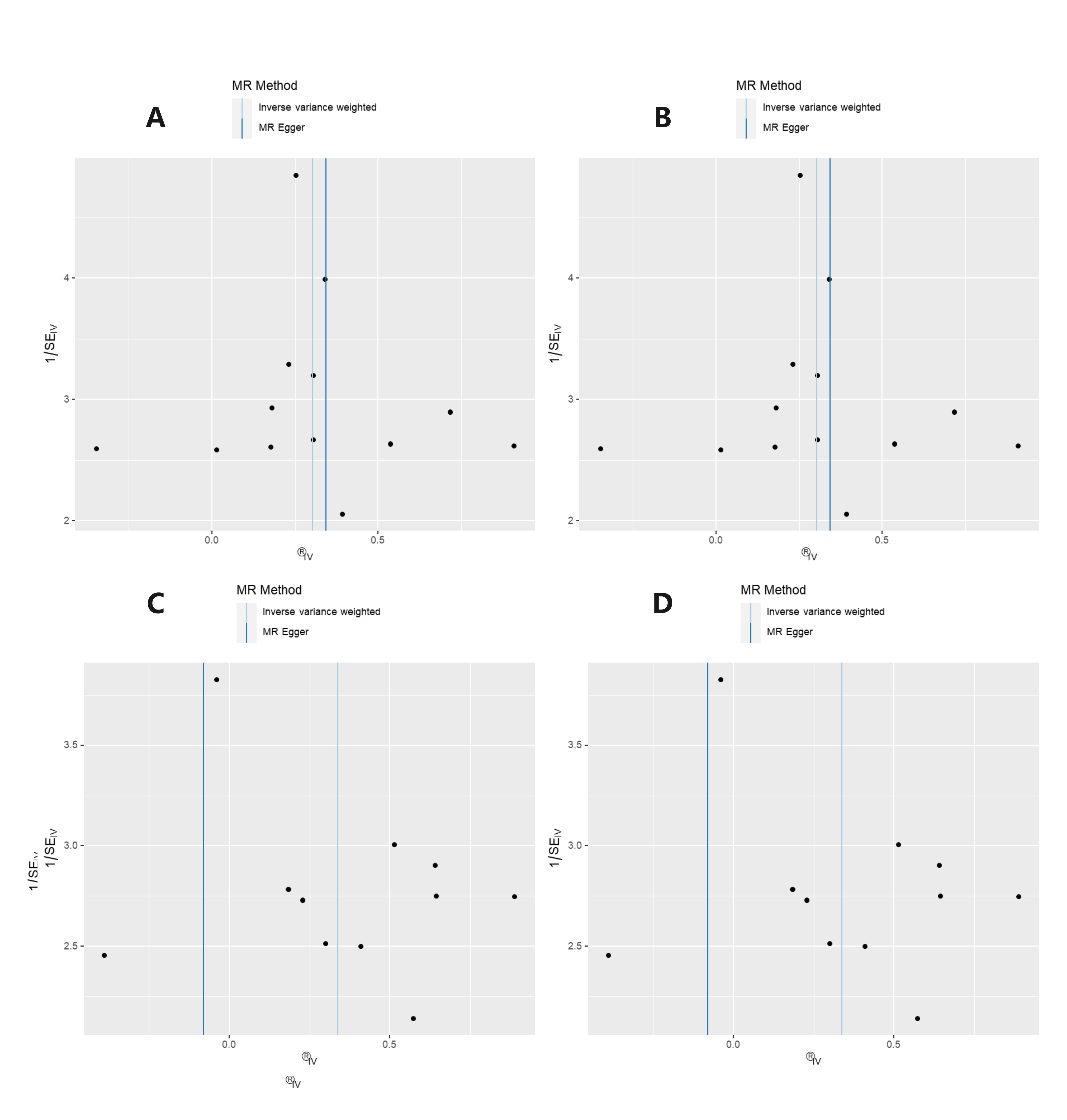

Supplement: Supplementary file 2 — Supplementary Figure 2: Funnel plots of the significant results: Funnel plots of the associations between the gut microbiota and six common complications of diabetes. A Bacteroidia class and diabetic hypoglycemia. B Bacteroidales order and diabetic hypoglycemia. C Verrucomicrobiae class and diabetic nephropathy. D Verrucomicrobiales order and diabetic nephropathy [file 13098_2024_1298_MOESM2_ESM.tif]
